# The Dangers of Electricity in Treatment

**Published:** 1912-11-23

**Authors:** 


### THE DANGERS OF ELECTRICITY IN TREATMENT.

### Address by Professor Jellinek, of Vienna.

At the meeting of the Section of Electro-Therapeutics of the Royal Society of Medicine on Friday, 15th inst., Professor Jellinek, of the Vienna University, gave an epidiascope and cinematograph demonstration on the subject "The Dangers of Electricity from the Clinical, Forensic, and Hygienic Points of View."

Professor Jellinek said that in Vienna fatalities had occurred from touching a conductor carrying only 100 volts; yet, on the other hand, in some cases where wires conveying a charge of 10,000 and even 20,000 volts had been touched the victims only suffered injury: they were not killed. To understand such a seeming contradiction regard must be had to all the circumstances—*i.e.* (1) external, (2) individual or personal. The first included voltage, amperage, number of poles touched, time during which the current passed. The second or individual circumstances embraced the resistance of the body, its general condition, the path taken by the current through the body, and whether or not the person or animal was accustomed to receive charges of electricity. In one case a man was killed by a current of only 65 volts; but another was not even rendered unconscious by coming into contact with 20,000 volts. In considering the body resistance, the skin was the most important structure, and the resistance offered by it varied with the thickness, hardness, and degree of moisture at the site where the shock was received, being least in a thin, moist skin. An important point was the condition of the floor on which the victim stood, whether carpeted, and to what degree it was insulated, and his proximity to earthed conductors, such as gas pipes. A prolific cause of accident was manipulating switches without proper insulating gloves. In Zurich a man caught in a broken telephone wire had his neck cut and burnt through by the live wire. Experiments in Vienna on the horse showed that when the current was applied it passed not only *via* the skin, but also through the body and brain. Horses and white mice were very susceptible to electric shock, tortoises and frogs scarcely at all; and a large series of experiments had convinced him that collapse from electric shock was really suspended animation, and the victims were amenable to restoration by first-aid methods, as in the case of the apparently drowned. And the first few minutes after the accident were of such paramount importance that if there were only one person on the spot at the time, his first effort should not be expended in fetching a doctor but in trying to restore the patient. The Electro-Patholgical Museum in Vienna had been of the utmost value in rightly judging cases of suspected murder, suicide, etc., by electricity, and in assessing evidence in legal cases, such as those pressing claims for compensation. A large number of lesions electrically produced were pictorially represented, and the Professor laid stress on the fact that such were practically always painless, so that complaint of pain by the appellant negatived electricity as the cause. Moreover, wounds produced electrically usually healed with a smooth, soft cicatrix. In conclusion, the Professor showed films kindly lent by the Austrian Minister of Labour, which were used to instruct artisans and labourers how to obviate danger when working in connection with electricity and the best means of resuscitation when their colleagues met with an accident.

Dr. H. Lewis Jones commented on the slight degree to which people who met with non-fatal accidents from electricity suffered from various nerve conditions. And in fatal cases the death seemed to be brought about chiefly by the serious thermal effects of the current. One case, which did not die at the time, was so badly burnt in the arms and legs that the muscles and vessels of the limbs formed a coagulum. The great variety in strength of current required to kill different people was one of the most peculiar aspects of the whole subject.

Professor Sylvanus Thomson, D.Sc., paid a high tribute to the care with which Government departments watched over, by what was often spoken of as "grandmotherly legislation," artisans in dangerous trades; and thought that this, together with the intellectuality of the workers themselves, was responsible for the remarkably few accidents in England in connection with switchboards. There would be even less if more regard were paid to the regulations.

Mr. Scott Ram (Electrical Inspector to the Factory Department at the Home Office) spoke highly of the Schäfer method* of resuscitation, and urged that doctors as a body should have a keener realisation of the recoverability by ordinary means of resuscitation of those who appeared to have died from electric shock. In one case which came under his cognisance, after the doctor had pronounced life extinct the victim's mates determined to "have another go at him," and succeeded in restoring him to life.

* This method and its relative advantages compared with the Silvester method was dealt with in The Hospital of July 11, 1908, page 379. Schäfer's system has been adopted by the Metropolitan Police as preferable to all others, as the result of investigations by the Royal Society of Medicine. The London County Council allows First Aid Classes to be instructed in the Schäfer method alone.

